# Histamine and motivation

**DOI:** 10.3389/fnsys.2012.00051

**Published:** 2012-07-04

**Authors:** Fernando Torrealba, Maria E. Riveros, Marco Contreras, Jose L. Valdes

**Affiliations:** ^1^Facultad de Ciencias Biológicas, Departamento de Fisiología, Pontificia Universidad Católica de ChileSantiago, Chile; ^2^Millenium Nucleus in stress and addiction, Pontificia Universidad Católica de ChileSantiago, Chile; ^3^Facultad de Medicina, Departamento de Fisiología y Biofísica, Instituto de Ciencias Biomédicas, Universidad de ChileSantiago, Chile

**Keywords:** addiction, apathy, appetite, histamine, infralimbic cortex, motivation, tuberomamillary nucleus

## Abstract

Brain histamine may affect a variety of different behavioral and physiological functions; however, its role in promoting wakefulness has overshadowed its other important functions. Here, we review evidence indicating that brain histamine plays a central role in motivation and emphasize its differential involvement in the appetitive and consummatory phases of motivated behaviors. We discuss the inputs that control histaminergic neurons of the tuberomamillary nucleus (TMN) of the hypothalamus, which determine the distinct role of these neurons in appetitive behavior, sleep/wake cycles, and food anticipatory responses. Moreover, we review evidence supporting the dysfunction of histaminergic neurons and the cortical input of histamine in regulating specific forms of decreased motivation (apathy). In addition, we discuss the relationship between the histamine system and drug addiction in the context of motivation.

## Introduction

Motivations are essential for the preservation of genes, and they provide a rational and structured explanation of behavior and brain organization. Normal and pathological appetites provide guidance; for example, they provide direction in decision-making and influence subsequent behavioral and physiological expression. The scientific study of behavior took an important step forward when the the concept of motivation (sometimes called instinctive behavior, drive or self-regulatory behavior) became widely accepted. It is important to understand why, of all the many possible behaviors in a given context, animals usually perform only a few or just one. In addition, animals in a specific context perform one action and, at other times in the same exact context, perform another action, which may sometimes be opposite to the former. A simple and fixed relationship between the stimulus and response is not a satisfactory model for the behavior of animals with a nervous system. Motivation can be affected by the internal state of the animal, and as other theorists have suggested, arousal or the energizing of behavior is a central component of motivation.

D. Hebb (Hebb, 1955) presented a model of motivation that is particularly useful for discussion; this work is accessible, along with other classic papers in Psychology, at http://psychclassics.yorku.ca/. Briefly, Hebb suggested that motivation provides direction and intensity for behavior; he was probably referring exclusively to higher vertebrates. Motivation is directed toward or away from reinforcers (a functional term for rewards, associated only with positive reinforcers). Positive reinforcers may attract appetitive behavior, while aversions drive behavior away from reinforcers. A historical account of the emergence of the concept of motivation is worth reading (Stellar, 1990).

An additional useful distinction that enriched the concept of motivated behaviors was proposed by Craig in 1917 (Craig, 1918); this distinction is a key element when considering the role of the brain histamine system in behavior. Craig suggested a distinction between the appetitive and consummatory phase of a motivated behavior. Craig stated that “an appetite, so far as externally observable, is a state of agitation which continues so long as a certain stimulus is absent. When the appeted stimulus is at length received it releases a consummatory reaction, after which the appetitive behavior ceases and is succeeded by a state of relative rest, a state of satisfaction.” This statement emphasizes that the arousal component characterizes only the appetitive phase of a motivated behavior.

These phases of motivated behavior have counterparts in the physiology of the somatic and visceral output systems that prepare the organism and allow it to better maintain bodily homeostasis and to perpetuate genes. The key role of behavior in long-term homeostatic balance and the preservation of genes has been thoughtfully addressed by Garcia (Garcia et al., 1974) and Blessing (Blessing, 1997), among others.

We will discuss the involvement of brain histamine in appetitive and aversive behaviors; in these behaviors, high arousal and an increased readiness to act and to spend energy predominate in parallel with neuroendocrine and sympathetic activation (Akins and Bealer, 1993). We will also argue that brain histamine may have a negative effect on consummatory behavior.

## Efferent connections of histaminergic neurons

In the mammalian brain, neuronal histamine is exclusively present in the tuberomamillary nucleus (TMN), a loosely packed set of magnocellular neurons located in the posterior and ventral region of the hypothalamus, in close proximity to the posterior recess of the third ventricle (Panula et al., 1989). Histaminergic axons innervate many brain regions, from the prefrontal cortex to the spinal cord, providing an excitatory tone to postsynaptic neurons through H1 and H2 receptors (H1R, H2R) and modulating the release of histamine and other transmitters through H3 receptors (H3R). This widespread distribution of histamine-containing axon terminals and histaminergic receptors helps to clarify the involvement of histamine in many brain functions. However, there have been indications that subsets of TMN neurons, some of which have a specific sensitivity to pharmacological agents, project to defined brain regions (Giannoni et al., 2009) or influence particular brain functions (Miklos and Kovacs, 2003; Valdes et al., 2010).

## What inputs drive TMN activity?

To begin unraveling the roles of histaminergic neurons in a variety of brain functions, it is important to consider the main inputs that might drive, by either increasing or decreasing the activity of TMN neurons. Driver inputs, as opposed to modulatory inputs, are those inputs that are central to the functions of a given cell, whereas modulators may change the expression of those functions (Sherman and Guillery, 1998); for example, a synaptic input may be either a driver or modulator depending on the conditions. We will show the importance of some of these inputs in defining the separate roles of histamine in wake/sleep cycles, feeding-related anticipatory activity, and motivation.

**Figure 1:**
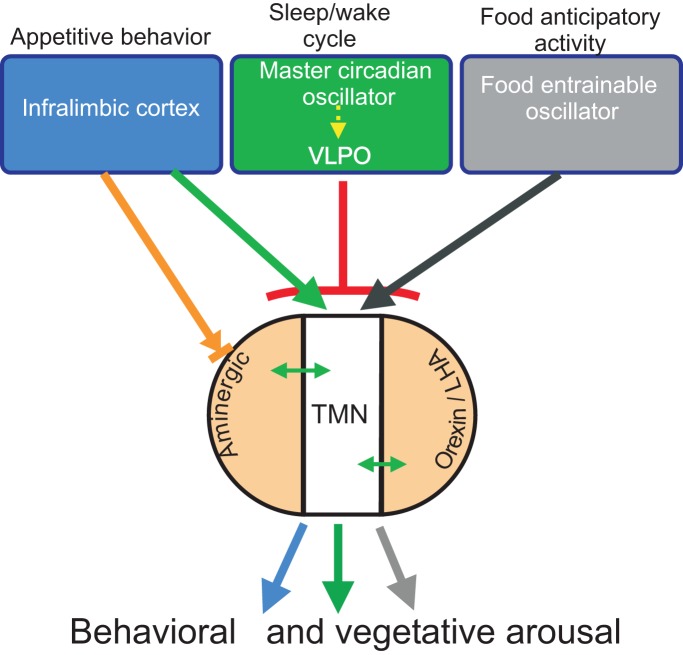
**Diagram of the putative drivers (blue, green, and gray) and modulatory (light orange) inputs to the TMN.** The presumed role of the driver inputs in the distinct arousal functions of the TMN is indicated on top; in brief, inputs from the prefrontal cortex or the food-entrainable oscillator activate the TMN during appetitive behavior or circadian anticipatory activity, respectively, while input from the VLPO inhibits TMN activity and promotes sleep. The green arrow indicates excitatory input; the black arrow designates presumed excitatory input; the red T-line indicates inhibitory input; the orange arrow designates mixed input to aminergic neurons. Double-headed arrows indicate bidirectional input between the TMN and aminergic and orexin neurons.

Neuroanatomical studies have addressed the origin of TMN afferents using axonal tract-tracing methods. In general, one can simplify the many inputs ascribed to the ventral TMN by focusing on the most robust (Ericson et al., 1991), considering that some of them may be driver inputs. TMN afferents originate from rostral forebrain limbic structures that include hypothalamic regions involved in food anticipatory activity (Acosta-Galvan et al., 2011), the infralimbic cortex (IL) (Wouterlood et al., 1987), the hypothalamic preoptic region and the lateral septum (Ericson et al., 1991; Sherin et al., 1998). Afferents from other aminergic nuclei and hypothalamic regions, including areas involved in circadian rhythmicity (Deurveilher and Semba, 2005) and the orexin/hypocretin neurons from the lateral/perifornical hypothalamic area, are likely modulatory rather than driver inputs (Ericson et al., 1989; Torrealba et al., 2003) (Figure 1).

## Food entrainable circadian oscillator input

**Figure 2:**
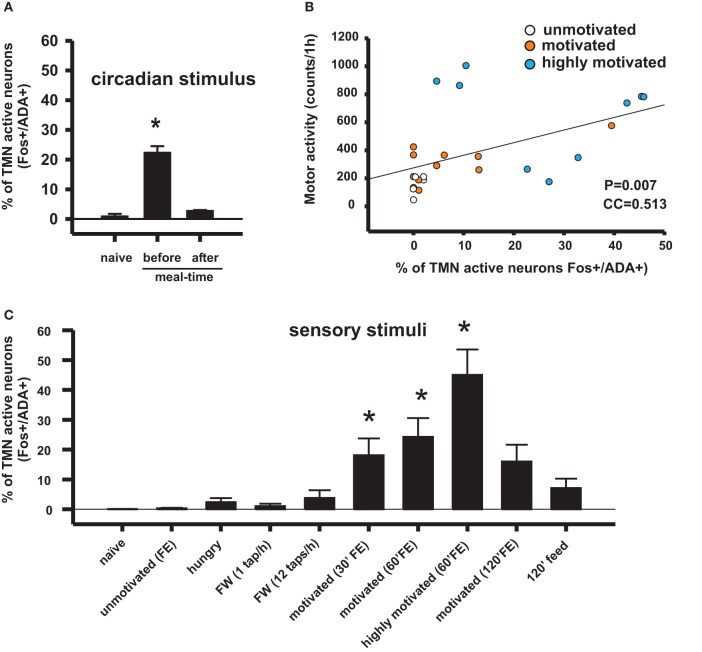
**Conditioned sensory signals and circadian signals can activate appetitive behavior and the TMN in rats, depending on the homeostatic state and the hedonic value of the sensory stimuli. (A)** TMN activation in anticipation of meal time in food-entrained rats disappeared after having the meal. Naive rats assessed at the same circadian time as rats assessed before meal time (Meynard et al., 2005). **(B)** Correlation coefficient between TMN activation (fos-ir) and goal-directed motor activity during enticing with food. This type of motor activity is used as an arousal index. The Pearson product moment correlation (Valdes et al., 2010). **(C)** Percentage of histaminergic neurons that became active (Fos-ir/ADA-ir) during different conditions that may induce arousal. Kruskal–Wallis One-Way ANOVA followed by multiple comparisons (Dunn's method) versus the naive group; ^*^p < 0.05. FE, food enticing; FW, forced wake induced by tapping the rat's cage.

The relationship between the motivation for food, arousal, and TMN activity was first studied using a model of restricted feeding. Rats placed on a feeding schedule that is restricted to a few daytime hours wake up in anticipation of mealtime. This anticipatory behavior has an important adaptive value because in nature, food may be available during the same few daily hours (Stephan, 2001), and the anticipatory physiological and behavioral activation prepares the animals to take advantage of this predictable phenomena. It was shown that this anticipatory waking up is related to transient activation of the TMN (Figure 2A) but not of other ascending arousal system (AAS) nuclei (Inzunza et al., 2000; Angeles-Castellanos et al., 2004), including the orexin neurons from the lateral hypothalamic/perifornical area (LHA) (Meynard et al., 2005). Increased TMN activation begins approximately one hour before the scheduled mealtime (Meynard et al., 2005), as evidenced by fos mRNA expression; however, the precise relationship between fos expression and electric activity of the TMN neurons remains to be determined. LHA neurons, including non-orexin neurons, become active well after TMN peak activation. We hypothesized that a signal from a food-entrainable circadian oscillator (Meynard et al., 2005) other than the suprachiasmatic nucleus (Mistlberger, 1994) should excite the TMN. Of interest for the present study is the distinction between the histaminergic effects on appetitive versus consummatory behavior. This anticipatory TMN activation quickly disappears (Figure 2C) when the animals begin eating (Meynard et al., 2005). Our preliminary data suggest that a bilateral TMN lesion impairs the anticipatory arousal induced by scheduled restricted feeding (Recabarren et al., 2003). Together, these pieces of evidences support the idea discussed that increased histamine levels are important for appetitive behavior and that decreased histamine levels facilitate consummatory behavior. It is likely that the food-entrained circadian input that activates the TMN arises from a component of the intrahypothalamic network that determines food anticipatory activity (Acosta-Galvan et al., 2011) and provides afferents to the TMN. The dorsomedial hypothalamic nucleus, subparaventricular zone, and medial preoptic area are possible candidates for this component (Deurveilher and Semba, 2005).

## The infralimbic cortical area input

**Figure 3:**
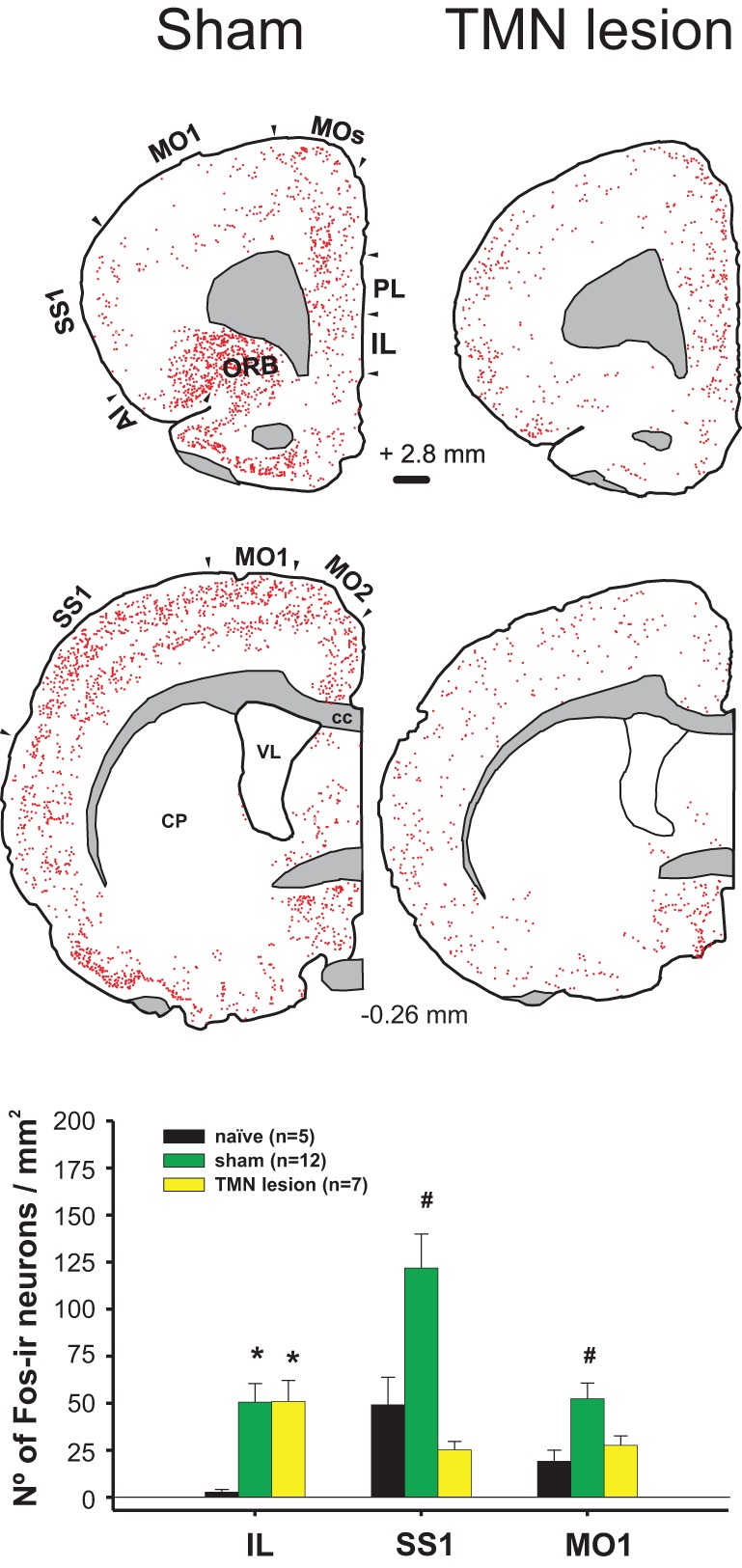
**Enticing intact (sham) hungry rats with food strongly activated the frontal cortex (left column).** This activation was impaired by a TMN lesion (right column), except for the IL. Lower graph compares the activation of three cortices in the sham and TMN lesioned rats. The naïve group corresponded to the circadian control, i.e., to rats fed *ad libitum*, not enticed and sacrificed at the same time during the day as the other groups (Valdes et al., 2006).

Exposure to appetitive food stimuli activates many brain regions in humans (Wang et al., 2004) and rats (Valdes et al., 2010). In both species, there is a marked activation of the frontal cortex, including the anterior insula and orbitofrontal cortices (Figure 3), both of which are closely involved in motivation and send projections to the LHA in rats (Gabbott et al., 2005). The activation of these frontal cortices was impaired by a TMN lesion (Valdes et al., 2010).

We view the cortical input to the TMN as essential for the appetitive function of histaminergic neurons, which will subsequently be discussed in more detail. Layer 5 pyramidal cells from the medial prefrontal cortex, which mostly originate from the IL, are the main cortical inputs to the TMN. The close proximity of the axon terminals from the IL to the histaminergic neurons and the glutamatergic phenotype of the pyramidal cells strongly suggest that IL afferents have an excitatory effect on histaminergic neurons. However, it is possible that the IL afferents may have an inhibitory effect if they make synaptic connections with local inhibitory neurons. An electron microscopic study elucidating the type of synaptic contact between the IL and TMN, which is presumably asymmetric and excitatory, similar to the other IL targets (Torrealba and Muller, 1999), is still lacking.

We reasoned that the prefrontal cortex, which is central in decision-making and motivation, is the input that activates the TMN during the appetitive procurement of reinforcers such as food. In fact, Goldman-Rakic demonstrated that the prefrontal cortex is the only source of cortical connections to arousal nuclei and that as such, the prefrontal cortex may control its own level of activity (GoldmanRakic, 1987). To study the functional relationship between IL and TMN neurons in motivated behavior, we devised a method to prolong the appetitive phase by enticing the rats with a wire-mesh box filled with food that was placed within their home cage. The rats may attempt to open the box, but they are unable to obtain the food [see Methods and video in Valdes et al. (Valdes et al., 2010)]. This procedure clearly separates appetitive from consummatory behavior.

Of particular relevance is that a subpopulation of rat IL neurons becomes active during enticement, as demonstrated in single unit recordings in freely moving animals and by c-fos expression (Valdes et al., 2006). A relatively large proportion, 33.3% of IL neurons, increase their firing rate during this enticing stage, while 10% become excited immediately after eating, and 3.3% became active in both conditions. A small percentage (6.7%) of IL neurons decreased their firing rate in response to enticement and after eating. This high proportion of IL neurons that responded to enticement suggests that the IL might use a population code to represent a given behavioral state. Taking into account the many specific functions of the IL during, for example, fear extinction (Quirk and Beer, 2006), stress (Amat et al., 2005), or enticement, and its numerous subcortical targets (Gabbott et al., 2005), one can imagine that subpopulations of IL neurons may participate in several of those behavioral states and that the global “visceral motor” IL (Terreberry and Neafsey, 1987) output may reflect such a combinatorial effect.

### IL-TMN axis-dependent mechanisms of behavioral responses to enticement

**Figure 4:**
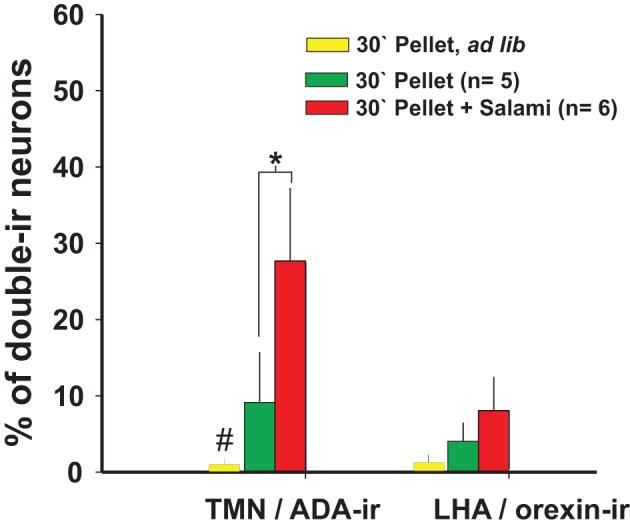
**The number of TMN active neurons that responded to enticing was dependent on the homeostatic state and palatability of the stimulus: Orexin neurons from the LHA did not respond to enticing.** ^#^Different from the other conditions; ^*^different from the pellet plus salami. Modified from Valdes et al. (2010).

The increased arousal state made apparent during enticement was measured by polysomnographic recordings (Valdes et al., 2005) or was evaluated by rat motor activity, which in this case and as observed in the video provided by Valdes et al. (Valdes et al., 2010), is goal-directed and not at all non-specific. Animals are motivated to obtain a reward when they detect homeostatic needs (hunger in the present example) and/or to obtain pleasure. One day of fasting ostensibly increased the rats' motivation to open the box more than feeding them *ad libitum*. Additionally, fasted rats familiar with a mixture of plain food pellets and palatable morsels (salami and chocolate cookies) made even more intense attempts to obtain the food compared to rats that were only offered plain pellets (Valdes et al., 2010) (Figure 2B). The increase in goal-directed motor activity, and therefore arousal, lasted an average of 30 min in the case of enticement with common pellets and >60 min in the case of enticement with salami and pellets, which was always accompanied by a proportional (in magnitude and duration) increase in the core temperature. These results showed that the enticement procedure used to assess motivation is sensitive to both the homeostatic and hedonic components of appetitive behavior. The use of early gene expression to evaluate the simultaneous activation of arousal nuclei revealed that enhanced motivation (either by hunger or by the anticipation of a more palatable food; Figure 2B) corresponded to increased activity of the TMN but not of the other arousal nuclei (Figures 2C and 4).

Whereas it is known that TMN activation during appetitive behavior depends on an intact IL (Valdes et al., 2006), it is still unclear whether the opposite is true. A TMN lesion (Valdes et al., 2010) decreased the activation of many cortical areas other than the IL (Figure 3). However, there is significant histaminergic innervation of the IL. Histaminergic axon terminals are present at a moderate density in the medial prefrontal cortex, including the IL (Panula et al., 1989), and histamine is released within those cortices (Westerink et al., 2002; Giannoni et al., 2009). Highly dense H1R ligand binding is present in the medial prefrontal cortex (Bouthenet et al., 1988), whereas H3R binding is present in the axon terminals of extrinsic origin; radiopharmaceutical ligand binding to H3R, but not H3R RNA, was expressed in the IL (Pillot et al., 2002). It has been shown that H3R activation in the medial prefrontal cortex decreases histamine release in both the TMN and prefrontal cortex of freely moving rats, which is a form of negative feedback (Flik et al., 2011). This finding may help to explain why TMN lesions, which should decrease histaminergic release in the prefrontal cortex, have little effect on IL activity during enticement. When using enticement as a model for appetitive behavior, we found that TMN lesions prevented increases in goal-directed motor activity (Valdes et al., 2010); this decreased appetitive behavior was correlated with the size of the lesion in the dorsal TMN. A bilateral IL lesion also impaired TMN activation during enticement (Valdes et al., 2006). Both the IL and TMN lesions blocked the delayed activation of other AAS nuclei, but not the locus coeruleus (LC) and orexin neurons, suggesting that these neurons contribute to arousal maintenance but not appetitive behavior. It is conceivable that the maintenance of IL activation during enticement after a TMN lesion might be the result of both decreased H3R inhibition and this spared LC activity and the minor activation of orexin neurons (Valdes et al., 2010). In fact, the IL receives a more substantial overlap of noradrenergic and orexinergic input (Baldo et al., 2003) compared with other cortical regions. It is possible that these two inputs maintain IL activity while the other cortical regions are depressed by TMN lesions.

**Figure 5:**
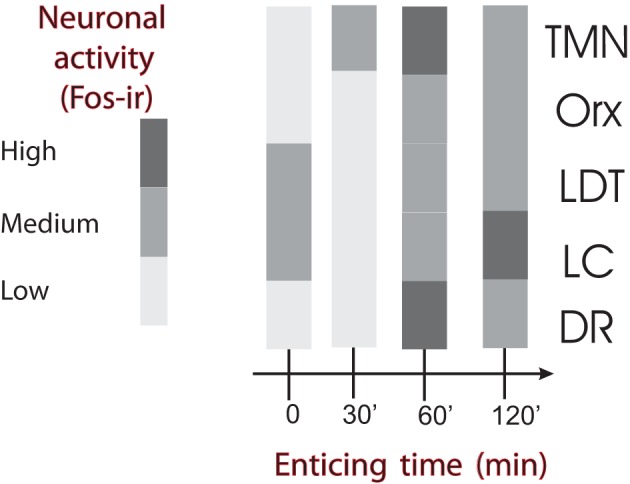
**Temporal course of ascending arousal system nuclei activation during appetitive behavior induced by enticing hungry rats with unreachable food.** The level of neuronal activity, evaluated by Fos expression, is indicated by the grayscale. The four bar graphs correspond to rats that have fasted for 24 h and were sacrificed immediately before the beginning of the enticing procedure (time 0) or killed after the indicated times (30–120 min). Note that the first nucleus to increase Fos-ir 30 min after the beginning of enticing was the TMN. The other arousal nuclei increased Fos-ir after 60 min and continued with medium to high Fos-ir at 120 min. DR, dorsal raphe; LC, locus coeruleus; LDT, laterodorsal tegmental nucleus; Orx, orexin neurons from the lateral/perifornical hypothalamic area; TMN, tuberomamillary nucleus. Modified from Valdes et al. (2005).

The IL, initially described as a visceral motor cortex (Terreberry and Neafsey, 1987), appears to be essential for the activation of the body and brain that takes place during the anticipation of an event and during appetitive behavior. The IL is a cortical area with more direct connections to subcortical sites involved in neuroendocrine and somatic responses related to appetitive or aversive behaviors (Gabbott et al., 2005). One of those responses is the arousal function of the IL during appetitive behavior, which is importantly mediated by its connection to the TMN. The TMN becomes active and increases arousal (Valdes et al., 2005) before the other AAS nuclei (Figure 5), and together with the IL, contributes to the delayed activation of the other AAS nuclei, which in turn maintain and potentially enrich the brain functions that optimize the expression of appetitive behaviors. For example, using the same behavioral task (Robbins and Everitt, 1995), it was shown that cortical cholinergic functions contribute to the accuracy of behavioral responses; these functions include the action of dopamine in delaying responses, the action of noradrenaline from the LC in distraction, and the action of serotonin that is related to rats' response impulsivity.

Novelty has reinforcing properties that motivate the exploration of new environments or novel objects. Evidence indicates that this exploration depends on an intact prefrontal cortex (Daffner et al., 2000) and histamine system. It has been shown that mice lacking the enzyme histidine decarboxylase (HDC) show decreased spatial novelty-induced arousal (Parmentier et al., 2002) and reduced exploratory activity in an open field but normal habituation to the same open field (Dere et al., 2004). However, another study found no differences between HDC-KO and wild-type mice in locomotor activity during a 2 h exposure to a new chamber (Nuutinen et al., 2010). Mice lacking H1R (Inoue et al., 1996) show reduced exploratory behavior (ambulation and rearings) in a novel environment. Furthermore, mice lacking H1Rs show reduced emotional responses to a novel environment and do not generate a place preference conditioned by the novel object; however, they do explore (consummatory behavior?) new objects in a familiar setting (Zlomuzica et al., 2008). The nucleus accumbens (NAcc) is one site where histamine increases exploratory behavior through the activation of H1 and H2Rs (Orofino et al., 1999). In contrast, histamine administration into the ventral hippocampus decreased exploration and associated emotionality in an H1- and H2-dependent manner (Ruarte et al., 1997). However, the existence of small regions in the NAcc that may increase or decrease the hedonic response (hotspots) (Smith and Berridge, 2007) makes it difficult to interpret the responses to a NAcc injection, whose precise target within this nucleus is unknown.

### IL-TMN axis-dependent mechanisms of vegetative responses to enticement

The complexity of the mechanisms underlying the vegetative arousal that characterizes appetitive behavior is illustrated by emphasizing the thermogenic response to enticement. A cell-specific lesion that involves more than 50% of the bilateral IL (Valdes et al., 2006) or a large TMN lesion (Valdes et al., 2010) prevents the thermogenic response to enticement. This effect was correlated with the size of a ventral but not a dorsal TMN lesion (Valdes et al., 2010). However, the normal pattern of thermoregulatory nucleus activation was differentially disrupted by IL versus TMN lesions. A lesion to the IL prevented the activation of all of the thermoregulatory nuclei examined, such as the preoptic area, dorsomedial nucleus of the hypothalamus, and raphe pallidus. In contrast, a TMN lesion only prevented the activation of the raphe pallidus (where sympathetic premotor neurons to brown adipose tissue are located). Other thermoregulatory nuclei were very active despite the absence of a thermogenic response. Importantly, while an IL lesion prevented TMN activation, a TMN lesion did not change IL activation during enticement. Taken together, these results indicate that the thermogenic response during appetitive behavior is controlled by the IL and TMN and that histaminergic input to the raphe pallidus plays a gating or permissive role in the IL thermogenic-related signals to the raphe pallidus. The temperature increase induced by activation of the IL-TMN axis is, in part, mediated by the action of histamine on H1Rs and H3Rs expressed by non-GABAergic and GABAergic neurons of the hypothalamic preoptic region (Lundius et al., 2010). This thermogenic response to histamine injection into the preoptic area is largely mediated by the activation of brown adipose tissue and its sympathetic innervation (Cannon and Nedergaard, 2004). Interestingly and similar to enticement, olfactory stimulation with grapefruit oil excited sympathetic innervation of the brown adipose tissue and adrenal gland and reduced the appetite for food. Moreover, all of these effects were blocked by the systemic administration of an H1 antagonist (Shen et al., 2005).

## Ventrolateral preoptic area input

The best-studied driver input to the TMN is the one central to the wake/sleep function of histaminergic neurons (Saper et al., 2005). This input arises from the ventrolateral preoptic area (VLPO) and adjacent regions in the anterior and ventral hypothalamus (Sherin et al., 1998). These neurons express the inhibitory transmitters GABA and galanin and make symmetrical GABA immunoreactive synaptic connections with the dendrites and cell bodies of TMN neurons and other neurons from the AAS (Sherin et al., 1998). VLPO cells are one of the few cell groups that are more active during sleep than wakefulness (Sherin et al., 1996). In addition, they inhibit AAS neurons, including the TMN, via GABA release, which results in sleep initiation and maintenance (Saper et al., 2005). This VLPO-TMN pathway is essential to understanding the well-described role of histaminergic neurons, which is to promote wakefulness during the active phase of the day.

The lateral septum is another source of robust, and mostly GABAergic, input to the TMN region. However, there are no published studies on the functional significance of this afferent connection. Nonetheless, histamine in the lateral septum has an anxiogenic effect that is mediated by H1 and H2Rs (Zarrindast et al., 2008).

## Orexinergic input

Orexin (hypocretin) neurons from the LHA, which are involved in appetitive behavior, in part because of their role in incentive saliency (Harris et al., 2005), form a well studied excitatory (Bayer et al., 2001; Eriksson et al., 2001) input to histaminergic neurons [while histamine has little effect on orexin neurons (Li et al., 2002)]. Orexin neurons may be considered a modulatory input to the TMN. For example, animals unable to synthesize orexin show intense electric activity of histaminergic neurons during cataplexia (John et al., 2004), indicating that inputs other than orexin maintain this high activity of histaminergic neurons. Orexin neurons have a variety of postulated roles, including arousal (Huang et al., 2001), in which histaminergic neurons appear to be the effector. Orexinergic neurons, perhaps under cortical influence (Monda et al., 2004), operate through type B receptors and histamine to increase brown adipose tissue sympathetic nerve activity (Yasuda et al., 2005) and thermogenesis.

## Histamine in aversive behavior

While the focus of this review is centered on the role of histamine in appetitive behaviors, it is useful to note that, in a more general sense, the role of histamine in the activation of the brain (behavioral arousal) and body (increased motor, neuroendocrine, and sympathetic activity) extends to behavioral changes associated with threat situations. Brown and colleagues (Brown et al., 2001) reviewed evidence supporting the idea that the brain histamine system is widely engaged in situations of physiological or existential danger. The anti-nociceptive action of central histamine and its role in water retention and adaptive anorexia highlight the relevance of the histaminergic system in situations of physiological threat, in which licking a wound, drinking, or eating is disadvantageous. For example, axon terminals expressing the hypothalamic anorectic peptide α-MSH, which perhaps originates in the arcuate nucleus, make synaptic contacts with TMN neurons (Fekete and Liposits, 2003) and may contribute to adaptive anorexia. Similarly, the histamine system is involved in situations of potential existential danger, i.e., stressful situations (Westerink et al., 2002; Miklos and Kovacs, 2003), which may elicit anxiety and a behavioral inhibition response (Gray and McNaughton, 2003) that is characterized by increased arousal, changes in behavioral focus, and increased exploratory behavior. For these reasons, Brown and colleagues proposed that brain histamine has a role as a danger response system. We offer a version of Brown's danger hypothesis that extends the range of goal-directed behavior from aversive to appetitive behavior. Histamine is released during goal-directed actions and results in an increase in behavioral and vegetative arousal and a decrease in the drive to consume, which allows the optimal progression of a motivated behavior. This short-lived adaptive anorexia is most likely an important feature of motivated behaviors.

## Histamine involvement in apathy

Reduced motivation or apathy has been defined as a reduction of self-generated, voluntary, and purposeful behaviors (Levy and Dubois, 2006). There are normal and pathological forms of apathy, depending on the cause, co-morbidity, and duration. Damage to the systems that generate and control voluntary actions located in the prefrontal cortex and basal ganglia will produce apathy. Apathy may be present in some forms of depression, dementia, schizophrenia, Huntington's or Alzheimer's disease, and frontal lobe injury, among others. In addition, apathy itself can also be a syndrome (Marin, 1991).

Levy and Dubois (Levy and Dubois, 2006) distinguish three types of pathological apathy based on the underlying psychological process and neural mechanism. (1) Emotional-affective apathy characterized by difficulties in behavioral modulation on the basis of emotional value, associated with damage to the orbitomedial prefrontal cortex, (2) cognitive apathy characterized by problems in planning and executing goal-directed behavior, associated with dysfunction of the lateral prefrontal cortex, and (3) auto-activation apathy characterized by the loss of spontaneity and a need of an external impulse to initiate actions, associated with injury to the medial dorsal prefrontal cortex, basal ganglia (internal globus pallidus), and/or paramedial thalamus. We think that the auto-activation type of apathy is characterized by a deficit in the energization of motivated behaviors, which we propose depend on an intact IL-TMN axis. Consistent with this idea, this type of apathy is caused by a lesion of the medial Brodmann areas 9/10, 24, 25, and 32; it is thought that the rat infralimbic cortical area is homologous to primate area 25 (Ongur and Price, 2000).

Drug abuse severity is associated with apathy scores (Verdejo-Garcia et al., 2006). Apathy is also associated with poor decision-making and the shortening of self-defined future in patients with frontal lobe damage (Fellows and Farah, 2005). The latter impairments may diminish the likelihood of success for psychological programs that manage drug addiction. Thus, treating apathy with methylphenidate (see below) might be beneficial in the treatment of addiction, as it has proven effective in weight-reducing programs (Desouza et al., 2011).

Interestingly, pathologies that exhibit co-morbidity with apathy have been characterized, from a neurochemical perspective, by dopaminergic (Bressan and Crippa, 2005) and histaminergic (see below) dysfunctions. However, none of these studies focused on the presence of apathy in the subjects analyzed; thus, the link between apathy and histaminergic or dopaminergic dysfunction is tenuous at present.

Regarding the histaminergic system, a reduction in H1R ligand binding in the frontal lobe of depressed patients (Kano et al., 2004) and schizophrenic patients (Iwabuchi et al., 2005) and the frontal and temporal regions of Alzheimer's disease patients (Higuchi et al., 2000) has been observed. However, chronic schizophrenic patients have increased levels of histamine metabolites in their cerebrospinal fluid (Prell et al., 1995). Parkinson's disease patients have increased levels of histamine but do not have increased levels of its metabolite, telemethylhistamine, in the putamen, substantia nigra compacta, and both divisions of the globus pallidus (Rinne et al., 2002); they also have increased histamine fibers in both divisions of the substantia nigra (Anichtchik et al., 2000). However, no increase in HDC mRNA expression was found in the TMN (Shan et al., 2012b) of Parkinson's disease patients, suggesting that there is no change in histamine production. Reduced H3R mRNA expression and increased histamine methyltransferase mRNA levels in the susbtantia nigra were also found in Parkinson's disease patients (Shan et al., 2012a). In Huntington's disease patients, HDC mRNA is increased in the inferior frontal gyrus and caudate nucleus, while H1 and H3R expression is increased in the inferior frontal gyrus and decreased in the caudate nucleus (van Wamelen et al., 2011). It remains to be determined whether these changes in the brain regions involved in appetitive behavior are a consequence of adaptations to the primary illness or whether they have a causal link to some of the symptoms common to these diseases.

Furthermore, it was found that apathy scores are higher in healthy males than in healthy females (cited in Verdejo-Garcia et al., 2006), while histamine H1R ligand binding is lower in the limbic system of males (Yoshizawa et al., 2009), which further suggests a link between brain histamine and motivation in human. In the same study, the authors found an increase in H1R ligand binding in the amygdala, putamen, and globus pallidus of anorexia nervosa patients (Yoshizawa et al., 2009). It is thought that anorexia nervosa patients are motivated to work around food (some may enjoy cooking, for example) but show a strong aversion to consume it. It was recently suggested that anorexia nervosa patients have a lower sensitivity to natural pleasurable reinforcers (not only to food), a trait that is modulated by cognitive processes focused on thinness (Soussignan et al., 2011).

Pharmacological evidence also indicates a causal relationship between histamine dysfunction and apathy. Methylphenidate strongly increases extracellular levels of dopamine, noradrenaline (Berridge et al., 2006), and histamine in the rat prefrontal cortex (Horner et al., 2007) and improves apathy scores in patients with Alzheimer's disease (Padala et al., 2010), stroke (Spiegel et al., 2009), and dementia (Dolder et al., 2010). It is possible that the increase in the histamine levels by methylphenidate is secondary to the increased extracellular concentration of dopamine, as is the case for systemic methamphetamine administration (Morisset et al., 2002), because D2 brain receptor activation enhances the TMN neuronal firing frequency, histamine release, and wakefulness in freely moving rats (Yanovsky et al., 2011).

We have shown that the IL activity and a consequent increase in histamine release and arousal are necessary for appetitive behavior. We, therefore, hypothesize that damage to the IL-TMN axis could induce a state of apathy that is characterized by a reduction in the disposition to work for rewards or avoid danger. In fact, conditions resulting in high apathy scores are associated with prefrontal damage linked to stroke, depression, or drug addiction (Verdejo-Garcia et al., 2006). The exploration of novel stimuli is a motivated behavior that is dependent on the histamine system (discussed above), which has been shown to be negatively correlated with high apathy scores in patients with Alzheimer's disease or frontal lobe damage (Daffner et al., 2000).

Anorexia may also be a normal response to a variety of physiological or pathological conditions. A well studied example is the reliable and phasic decrease in food intake that follows a cyclic increase in estrogen in rodents and primates, including humans (Geary et al., 2001). While several hypothalamic nuclei, including the TMN, express estrogen receptors, the anorectic effect of estradiol appears to depend on its direct action on TMN neurons, in addition to the effects of corticotropin-releasing hormone on TMN neurons (Gotoh et al., 2005). Estradiol also acts on the paraventricular hypothalamic nucleus (an important anorexigenic region), which releases corticotropin-releasing hormone (Gotoh et al., 2009). Histaminergic neurons may act on ventromedial hypothalamic nucleus (VMH) neurons via H1R to decrease food intake (King, 2006). Female hamsters show changes in brain histamine content following the estrous cycle (Hine et al., 1986); the highest level is reached on proestrous day (day 3) in the hypothalamus and on day 2 in the rest of the brain. During pregnancy, there was an overall decline in histamine content and an increase in food intake. Female rats and humans are more vulnerable to drug abuse in general but particularly on the days with higher estrogen levels (Anker and Carroll, 2011). This vulnerability is facilitated by estrogens.

The site of brain histamine-mediated suppression of food intake is likely the VMH (Ookuma et al., 1989; Malick et al., 2001; King, 2006). The VMH, a hypothalamic site that contains glucose-responsive neurons and descending axonal projections to hindbrain regions that contain premotor sympathetic neurons, is the somatomotor center for motivated behavior-related activities (Simerly, 2004). Blockade of H1R within the VMH, but not in other hypothalamic nuclei such as the paraventricular hypothalamic nucleus or the LHA, increases both meal size and duration and suppresses the activity of glucose-responsive neurons (Fukagawa et al., 1989).

## Histamine involvement in addiction

Addiction is a chronic behavioral disorder characterized by a compulsive and relapsing pattern of drug-seeking and drug-taking behavior that occurs despite the awareness of serious negative consequences. Thus, the behavior and cognitive processes of addicts are centered on drugs, and they have enormous difficulties in attending to other activities (Everitt and Robbins, 2005; Hyman et al., 2006).

Addiction develops after a prolonged period of drug intake in vulnerable individuals (Deroche-Gamonet et al., 2004; Vanderschuren and Everitt, 2004) and appears to depend on persistent neuroplastic changes (Kalivas and O'Brien, 2008) that include impairment in the synaptic plasticity of relevant brain circuits such as the NAcc (Kasanetz et al., 2010). Synaptic plasticity (long-term depression) at cortico-striatal synapses is critically controlled by dopamine (Surmeier et al., 2007) and acetylcholine (Wang et al., 2006), and it is thought to underlie the formation of goal-directed behaviors and habits.

It has been proposed that, as with habit formation in general, addiction involves a progression from action-outcome to stimulus-response mechanisms; drug use in the addict is controlled by automatic action patterns interacting with non-automatic cognitive processes (Tiffany, 1990; Everitt and Robbins, 2005; Pierce and Vanderschuren, 2010). This sequence, from behavior controlled by outcome to habitual drug seeking, is paralleled by the gradual recruitment of ventral to dorsal striatal regions (Porrino et al., 2002) that have an anatomical substrate in the spiraling connections between the medial prefrontal cortex, NAcc, and ventral tegmental area (VTA) and the dorsal prefrontal cortex, dorsal striatum, and substantia nigra (Haber et al., 2000; Pierce and Vanderschuren, 2010). The histaminergic system sends projections to the prefrontal cortex and striatum (Panula et al., 1989; Giannoni et al., 2009) and receives inputs from the medial prefrontal cortex, particularly from the IL. This cortical input drives TMN activity and seems to be important in sustaining a high level of arousal during motivated behavior, as discussed above. Because histaminergic axon terminals are present in the prefrontal cortex, dorsal striatum, and NAcc, it is possible that histamine may participate in the initial and late phases of addiction. Consistent with the idea presented in this review, histamine would amplify the incentive salience (Berridge and Robinson, 1998) of reinforcers and reinforcer-associated cues and support the arousal of the appetitive phase of motivated behavior directed toward obtaining drugs.

A link between the activity of the histamine system and the risk of becoming addicted to drugs has been reported. Novelty seeking is a personality trait that is related to an increased risk of addictive behavior in human and animal models (Kampov-Polevoy et al., 2004; Belin et al., 2011). Females have higher levels of brain histamine (Hine et al., 1986; Prell and Green, 1991) and are more vulnerable to addiction than males. High gonadal hormone estrogen levels during the menstrual cycle may facilitate drug abuse in women (Anker and Carroll, 2011). Anorexia nervosa patients show a significantly higher density of histamine H1R than controls, suggesting that the alteration of central histaminergic activity is involved in eating disorders (Yoshizawa et al., 2009). Interestingly, there is a co-morbidity between bulimia nervosa or anorexia nervosa and addiction (Baker et al., 2010). Rats with a strong preference for alcohol have elevated levels of brain histamine and its metabolites, as well as a higher density of histaminergic nerve fibers than rats with a lower preference for alcohol (Lintunen et al., 2001).

Despite these correlation studies, a clear association between histamine activity and the likelihood of engaging in drug seeking behavior is still lacking. The systemic administration of thioperamide to normal mice, which increases the firing of TMN neurons and histamine release in the prefrontal cortex and posterior hypothalamus (Flik et al., 2011) is effective in facilitating the development of conditioned place preference induced by a small (but not a larger) dose of cocaine (Brabant et al., 2005). However, thioperamide interferes with psychostimulant metabolism and maintains higher concentrations (Brabant et al., 2009). In addition, antagonizing H3R alters the release of other neurotransmitters through heterosynaptic mechanisms, making it difficult to ascribe its effects to histamine transmission only. Histamine inhibits the development of morphine-induced conditioned place preference (Gong et al., 2007, 2010); however, HDC-KO mice are not more responsive to the stimulant effect of cocaine and require cocaine doses similar to those required by WT mice to develop conditioned place preference (Brabant et al., 2007).

Drugs that are abused disrupt the neural circuitry involved in motivational processes such as pleasure, incentive saliency, and learning (Robinson and Berridge, 2008). The mesocorticolimbic dopaminergic system arising from the VTA and substantia nigra compacta is important in acquiring a conditioned inclination to stimuli that have been associated with obtaining primary reinforcers and in maintaining habits once a motivation-related task has been learned (Wise, 2004). Addictive drugs share the property of activating this mesocorticolimbic dopaminergic system, and the increase in dopaminergic concentration in the NAcc and frontal cortex appears to be an essential mechanism of drug addiction (Roberts et al., 1980; Koob, 1992). The frontal cortex and NAcc receive input from limbic structures and brainstem autonomic centers related to affective and motivational function (Kelley, 2004). The NAcc is a key region involved in the processing of stimuli reinforcement information and in the selection of the appropriate motor action toward the selected goal (Kelley, 2004; Salamone et al., 2007). Dopaminergic transmission in the NAcc appears to be important for assigning incentive salience to rewards and to conditioned stimulus-reward associations but seems to be irrelevant to hedonic processes (Smith et al., 2011). Brain histamine can also affect the neural operation of structures involved in motivation/reward processes and influence the mesocorticolimbic dopamine system in opposite ways by acting at different levels.

The nuclei of origin of the mesocorticolimbic system (VTA and substantia nigra compacta) receive high to moderate histaminergic fibers (Panula et al., 1989). H1R are present in the VTA and substantia nigra compacta (Bouthenet et al., 1988). In addition, H2R mRNA and protein are present (Vizuete et al., 1997) in the VTA and substantia nigra compacta at moderate levels. H3R mRNA is expressed in substantia nigra compacta but not in VTA neurons (Pillot et al., 2002), suggesting a presynaptic effect of histamine on nigrostriatal but not on VTA axon terminals in the NAcc or the prefrontal cortex. Histamine inhibits the activity of these dopaminergic neurons indirectly via the excitation of GABAergic neurons (Korotkova et al., 2002), although it is not known whether those GABAergic neurons are local or projecting.

The NAcc has a moderate number of histaminergic fibers (Panula et al., 1989) and high densities of H1, H2, and H3R (Bouthenet et al., 1988; Vizuete et al., 1997; Pillot et al., 2002), suggesting that histamine has the potential to exert complex effects on NAcc function but exhibits no effect on VTA dopaminergic terminals, which lack H3R. Intracerebroventricular (icv) administration of histamine stimulates mesolimbic dopamine neurons projecting to the NAcc through an action on H1R, while no such effect was found in the dorsal striatum (Fleckenstein et al., 1993). Local administration of histamine into the NAcc increases or decreases the firing rate of the accumbens neurons (Shoblock and O'Donnell, 2000) and increases local extracellular dopamine via H1 activation of cholinergic interneurons (Prast et al., 1999), which act on presynaptic nicotinic receptors to increase dopamine release (Wonnacott et al., 2000; Galosi et al., 2001). In addition, acetylcholine facilitates the inhibition provided by fast-spiking GABAergic interneurons on medium spiny neurons (MSNs) by activating postsynaptic nicotinic receptors (de Rover et al., 2002).

The dorsal striatum (caudate-putamen) receives a low to moderate density of histaminergic fibers (Panula et al., 1989) and contains high densities of H2R and H3R (Vizuete et al., 1997; Pillot et al., 2002) and a moderate density of H1R (Bouthenet et al., 1988). The substantia nigra sends projections to the dorsal striatum (Beckstead et al., 1979), receives a moderate density of histaminergic fibers (Panula et al., 1989) and has a high density of H3R, particularly in the pars reticulata (Pillot et al., 2002). Histamine H3R are located on nigrostriatal terminals, and their activation inhibits striatal dopamine synthesis (Molina-Hernandez et al., 2000). Histamine excites dissociated cholinergic striatal interneurons through H1 and H2R, which subsequently excite the MSNs, most likely via muscarinic receptors. In contrast, histamine has no direct effect on dissociated MSNs (Munakata and Akaike, 1994). In anesthetized rats, the local iontophoretic injection of histamine into the striatum increased the firing rate in 40% of the neurons (Sittig and Davidowa, 2001). Using a slice preparation from the dorsal striatum of mice, bath-applied histamine depolarized MSNs by acting on H2R and suppressed both cortical (Doreulee et al., 2001; Ellender et al., 2011) and thalamic excitatory inputs (studied by single-pulse stimulation) to MSNs acting on presynaptic H3R (Ellender et al., 2011). Histaminergic depression of excitatory input to the MSNs through H3R can also result from inhibition of glutamatergic release (Brown and Haas, 1999). Interestingly, histamine facilitates the short-term dynamics (studied by paired-pulse stimulation) of thalamostriatal more than the dynamics of corticostriatal synapses, leading to a larger facilitation of thalamic input in this condition (Ellender et al., 2011). Histamine has no effect on fast-spiking GABAergic interneurons but abolishes lateral inhibition between MSNs. Histamine also depolarizes cholinergic interneurons acting at H1Rs in the dorsal striatum (Bell et al., 2000), which increases MSN inhibition, as has been described in the NAcc. These results suggest that increased activity of the histaminergic system in the striatum in general may lead to an amplification of the feed-forward inhibition by interneurons on projection neurons and a facilitated response to thalamostriatal input.

The evidence presented here suggests that the histaminergic system contributes to the regulation of the normal function of the dorsal striatum and highlights the possibility that histamine may exert control over the behavioral and motor output of this region. The effects of histamine on the dorsal striatum suggest that histamine has a role in the later stages of addiction (Everitt and Robbins, 2005), which is when drug intake is driven by habit. While the mechanisms of action are not entirely clear and may influence the intake of specific drugs instead of interfering with addiction in general, it is interesting that pyrilamine significantly reduces nicotine self-administration (Levin et al., 2011) and that H3R antagonists reduce alcohol intake (Lintunen et al., 2001; Galici et al., 2011) and methamphetamine self-administration (Munzar et al., 2004) in animal models of addiction.

Aminergic neurotransmitters act on postsynaptic structures mostly through volume transmission, resulting in longer-lasting and more widespread effects (Torrealba and Carrasco, 2004) compared to fast neurotransmitters. Fast-scan cyclic voltammetry studies report that the half-life in the extracellular space of histamine released by electrical stimulation of the medial forebrain bundle is 4.1 ± 0.9 s (Hashemi et al., 2011). Similar studies have determined that the dopaminergic half-life is 1.12 ± 0.05 s (Park et al., 2010). No high-affinity uptake system for histamine has been reported; the termination of histamine synaptic action appears to require its catabolism to telemethylhistamine by the enzyme histamine N-methyl transferase in the extracellular space (Haas and Panula, 2003). Taken together, these findings suggest that in physiological conditions, histamine signaling could bring about longer-lasting effects compared to dopamine in the same brain region (i.e., NAcc shell), which could contribute to dopaminergic action on incentive salience and learning.

The data reviewed here suggest that the histaminergic system is involved in the motivation for reward and reward-associated stimuli that are relevant to the neural process that mediate addiction through the amplification of the physiological effects of dopamine and by its direct effects on the dorsal striatum and NAcc. However, the functional relationship between the histaminergic and the dopaminergic systems have not been studied in detail, in part because we lack of a clear conceptual framework of the links between the parallel and convergent functions of these two systems in motivated behavior.

## Conclusions

The brain histaminergic system is essential for the appetitive and the aversive phases of motivated behaviors. Such behaviors include the maintenance of homeostatic balance, the exploration of new environments, reproduction and caring of the progeny, and responses to threatening situations. The brain histaminergic system also contributes to successful goal-directed behaviors by decreasing the drive to consume. In addition, dysfunction of the histamine system may underlie some forms of apathy and feeding disorders. Finally, the histaminergic system may have a potentially important function in abnormal appetites for drugs, although this role has not yet been explored in detail.

### Conflict of interest statement

The authors declare that the research was conducted in the absence of any commercial or financial relationships that could be construed as a potential conflict of interest.
